# Mutational Analysis of the TYR and OCA2 Genes in Four Chinese Families with Oculocutaneous Albinism

**DOI:** 10.1371/journal.pone.0125651

**Published:** 2015-04-28

**Authors:** Yun Wang, Zhi Wang, Mengping Chen, Ning Fan, Jie Yang, Lu Liu, Ying Wang, Xuyang Liu

**Affiliations:** 1 Shenzhen Key Laboratory of Ophthalmology, Shenzhen Eye Hospital, Jinan University, Shenzhen, P. R. China; 2 Institutes of Translational Medicine, Nanchang University, Nanchang, P. R. China; 3 Department of Ophthalmology, the Second People's Hospital of Zhengzhou, Zhengzhou, P. R. China; Naval Research Laboratory, UNITED STATES

## Abstract

**Background:**

Oculocutaneous albinism (OCA) is an autosomal recessive disorder. The most common type OCA1 and OCA2 are caused by homozygous or compound heterozygous mutations in the tyrosinase gene (*TYR*) and *OCA2* gene, respectively.

**Objective:**

The purpose of this study was to evaluate the molecular basis of oculocutaneous albinism in four Chinese families.

**Patients and Methods:**

Four non-consanguineous OCA families were included in the study. The *TYR* and *OCA2* genes of all individuals were amplified by polymerase chain reaction (PCR), sequenced and compared with a reference database.

**Results:**

Four patients with a diagnosis of oculocutaneous albinism, presented with milky skin, white or light brown hair and nystagmus. Genetic analyses demonstrated that patient A was compound heterozygous for c.1037-7T.A, c.1037-10_11delTT and c.1114delG mutations in the *TYR* gene; patient B was heterozygous for c.593C>T and c.1426A>G mutations in the *OCA2* gene, patients C and D were compound heterozygous mutations in the *TYR* gene (c.549_550delGT and c.896G>A, c.832C>T and c.985T>C, respectively). The heterozygous c.549_550delGT and c.1114delG alleles in the *TYR* gene were two novel mutations. Interestingly, heterozygous members in these pedigrees who carried c.1114delG mutations in the *TYR* gene or c.1426A>G mutations in the *OCA2* gene presented with blond or brown hair and pale skin, but no ocular disorders when they were born; the skin of these patients accumulated pigment over time and with sun exposure.

**Conclusion:**

This study expands the mutation spectrum of oculocutaneous albinism. It is the first time, to the best of our knowledge, to report that c.549_550delGT and c.1114delG mutations in the *TYR* gene were associated with OCA. The two mutations (c.1114delG in the *TYR* gene and c.1426A>G in the *OCA2* gene) may be responsible for partial clinical manifestations of OCA.

## Introduction

Oculocutaneous albinism (OCA) is a group of autosomal recessive disorders characterized by hypopigmentation of the skin, hair and eyes, and is often associated with ocular changes including photophobia, decreased visual acuity and nystagmus[1]. The prevalence of OCA and its subtypes differs widely among different populations and it is approximately 1:18,000 in the Chinese Han population of Shangdong Province[2]. OCA is classified into seven types based on the different causative genes involved. OCA1 (MIM 203100), caused by homozygous or compound heterozygous mutations in the tyrosinase gene (*TYR*)[3, 4]on chromosome 11q14, is the most common type worldwide, with a prevalence of 1 per 40,000 individuals in most populations[5]. The OCA1A subtype is the most severe type which presents with white hair and milky skin throughout life, and has a complete lack of tyrosinase activity[4, 5] whereas type 1B (OCA1B), characterized by reduced activity of tyrosinase may develop some pigment with time[4, 5]. OCA2 (MIM 203200), OCA3 (MIM 203290), and OCA4 (MIM 606574) are somewhat milder forms of the disorder and are caused by mutations in the *OCA2* (previously called *P*), tyrosinase-related protein 1 (*TYRP1*), and *SLC45A2* genes (solute carrier family 45 member 2, previously called *MATP*) respectively. Recently, three additional types of OCA (OCA5, OCA6 and OCA7) have been identified[6]. By linkage analysis, OCA5 has been mapped to the human chromosome 4q24[7] and further studies are required to eventually identify the disease-caused gene. Two new genes (*SLC24A5* and *C10orf11*) have been shown to be the cause of OCA6[8] and OCA7 respectively[9]. In addition, there are several syndromic OCA genes which associated with Hermansky-Pudlak syndrome (*HPS 1–7*)[10], Chediak-Higashi syndrome(*LYST*) [11], and Griscelli syndrome types 1 *(MYO5A*)[12] and 2 (*RAB27A*) [13]. Mutations and variants of OCA are listed in the Albinism database (http://albinismdb.med.umn.edu/).

As mutations in the *TYR* and *OCA2* genes account for the majority of OCA cases, we have analyzed and examined the *TYR* and *OCA2* genes in four Chinese families with oculocutaneous albinism in the present study, identifying the causative mutations in each.

## Patients and Methods

### Patients

Four non-consanguineous OCA patients and 105 unaffected individuals were recruited in Shenzhen Eye Hospital. Written informed consent for genetic tests and publication of personal photographs was obtained from the guardians of the probands according to the principles of Declaration of Helsinki (see attachment for details). All 105 individuals in the control group were healthy and with no family history of albinism. This study was approved by Institute Review Board of Shenzhen Eye Hospital.

### Clinical examination

Complete physical examination and detailed ophthalmic examination were carried out on these four individuals. The following clinical features were recorded: varying colors of the skin and hair, and abnormal ophthalmological findings including photophobia, nystagmus and reduced visual acuity.

### Mutational analysis of the *TYR* and *OCA2* genes

Genomic DNA was extracted from 200μl of peripheral blood using the QIAamp DNA blood mini kit (QIAGEN, Hilden, Germany) by standard protocols. DNA integrity was evaluated by 1% agarose gel electrophoresis. The *TYR* and *OCA2* genes were amplified by polymerase chain reaction (PCR) and sequenced directly. PCR primers were designed by Primer Premier 5.0 which covered the sequences of all five coding exons of the *TYR* gene (Table 1) and 2–24 exons of the *OCA2* gene (Table 2). The primers were synthesized by BGI (BGI-Shenzhen, Guangdong, China). Each 30 μl PCR reaction mixture contained less than 1μg genomic DNA, 1.0 μM of each of the forward and reverse primers and 15 μl of 2×Taq PCR MasterMix containing 0.1U Taq DNA polymerase/μl, 500μM dNTPs, 20 mM Tris-HCl(pH8.3), 100 mM KCl, 3 mM MgCl_2_, PCR reaction enhancer, optimizer and stabilizer (Tiangen Biotech, Beijing, China). PCRs were carried out in a MyCycler thermocycler (Bio-Rad, Hercules, CA, USA) using the following steps: initial denaturation at 94°C for 2 min followed by 35 cycles of denaturation at 94°C for 10s, annealing at 55–59°C for 30s, and extension at 72°C for 45s, and then a final extension at 72°C for 5 min. The amplified products were purified with a cycle-pure kit (OMEGA; Bio-Tek, Doraville, GA) and sequenced using an ABI 377XL automated DNA sequencer (Applied Biosystems, Foster City, CA). The DNAStar (Madison, WI) software was used for DNA sequences assembly and analysis with a genomic reference sequence. The sequence variants were named according to the nomenclature recommended by the Human Genomic Variation Society (HGVS). To evaluate whether novel variants were predisposing mutations or polymorphisms, Sanger sequencing was performed on the corresponding region of the 105 control individuals.

**Table 1:** Primers used in PCR for amplification of *TYR*.

Exon	Sequence (5′→3′)		Annealing temperature(°C)	Product size(bp)
	Forward	Reverse		
1	TGGAGGTGGGAGTGGTATTA	GGCACCATTTCTGTCCTTGA	55	1072
2	CCAACATTTCTGCCTTCTCC	GTCACAGCCTCCCAAGTAAA	55	536
3	GGCTCAACCTCTTTTACCTG	GCCTAAGGTCTGTTTTGGTG	55	544
4	GCAGCATTCTGGAGGTTCAA	GGAATAACATTGTCGAAGCA	59	558
5	GGAATAACATTGTCGAAGCA	GAGTTGGAAGAAGGCTACAC	55	482

**Table 2:** Primers used in PCR for amplification of *OCA2*.

Exon	Sequence (5′→3′)		Annealing temperature(°C)	Product size(bp)
	Forward	Reverse		
2	TTGTACGGACTCCCAAGGTG	ATGCCCTCAGAGACATACGC	55	635
3	TGAGTTGGGGAGAAGAGGTC	AGCATCTCAGCCCTCAGTGT	55	645
4	AAAGACCAGGGTTGATTCGG	GCCTGAAGCAAACAGATCCT	55	614
5	TGGAAGTTACTCAAGGCTGC	TGAACAGGGAAGTGGTAAGC	55	463
6	CACACAGTAGCCCCATCATC	GTCACGCTGAACGCAAAAGA	55	501
7	TGAGGGCTGAGATGTGGGTA	CTGTGGCTCCCCATCAAATC	55	437
8	AAATTCCCAGCTCCAACCAC	TCTCACATCCTGACACCCAT	55	471
9	TGTTCATTGTCGGGTGGTGT	GAGAGAGGGACACGCTAATC	55	548
10	CGCTGCTTTATCAGGGTCAT	CACGATGAGGAATCACACCA	55	546
11	TCCACTCGGAATGTAACCAG	TCATCGTCAGACACGCCTTG	55	572
12	CAGATGGGATGGTAATGTGC	ACGATTCAACCTGAGTACCC	55	566
13	ATGGCATTACGGGGACTGAG	GCCTATGTCTTCCACCTCCT	55	665
14	GAGGCTCACTCTGGAAAGGA	GACCAGTCACCTAACATCCC	55	521
15	GTGTTAGCCAGGATGGTCTC	TGTGCTGGGTTATGTTGCTG	53	634
16	TCTCCTAATGCTATCCCTGC	GAGCCTCTGGTTTTGTGTTG	55	719
17	TCCAGCCAACAAATGAAGCC	CCTCCGCAAAGTGTGAACCT	55	555
18	CATCCTCGTGAAATCTGTCG	GGAAACCAAAAGGAAAGGCG	55	393
19	TGTGGGGGTTGACAAGAGGA	GTGGCTAAGGTAAAGCTGGT	55	569
20	CTGCCACTGCTGTTGGACTT	GGTTCCCTGCTGTGCCTTTT	55	405
21	CTATGTCTGCCTTGGTCTCG	CTCTGCTCACTTTCGTCCTC	55	379
22	ACTCTGTGTGCCTTTACCCC	GCATCAGAACCAGTCATCCC	55	610
23	GACTTGCCCATTCTCCTTAG	TCCCCTACACCACAGTCTCT	59	496
24	AGGTGCTAAGGCCATGTTCT	TTCTGGAGGGGAATCTTGAG	55	444

## Results

### Clinical phenotype

**Fig 1:**
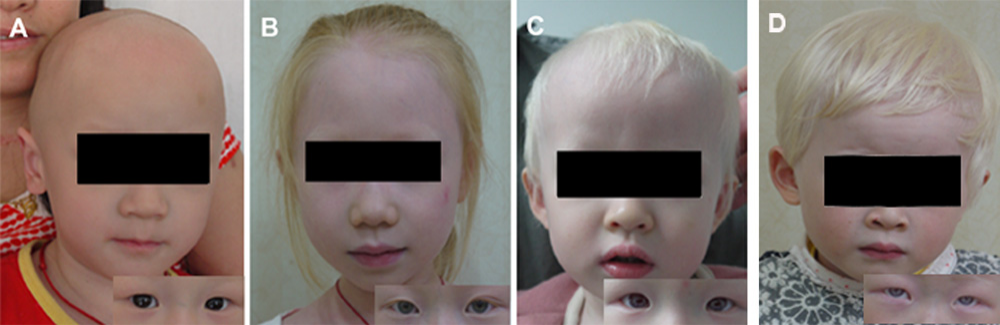
Clinical features of four OCA families. All the patients had typical OCA symptoms on the color of skin, hair and iris. Consent for these photos was obtained from the guardians of the probands.

Clinical features and mutational alleles of the four OCA patients are shown in Fig 1 and Table 3. All patients had typical OCA symptoms on their skin, hair and iris. They lacked pigment at birth but developed darker hair over the course of physical development, indicating an accumulation of pigment with time. Interestingly, some ophthalmically unaffected individuals in these two OCA families (family A and B) presented with blond or brown hair and milky white skin, and showed minimal to moderate pigmentation gradually accumulating with age, but with no manifestation of ocular anomalies.

**Table 3:** Clinical features and mutations for four Chinese patients of Oculocutaneous albinism.

Patient	gender	Age	Clinical features				Gene	Mutations		Diagnosis
		(year)	Hair color	Skin color	Iris color	Nystagmus		Paternal	Maternal	
A	M	4	White	Milky	Brown	Positive	*TYR*	c.1114delG (p.G372fsX112) *	c.1037-7T.A, c.1037-10_11delTT	OCA1
B	F	8	Blond	White	Green	Positive	*OCA2*	c.593C>T(p.P198L)	c.1426A>G(p.N476D)	OCA2
C	F	2	White	White	Pink	Positive	*TYR*	c.896G>A(p.R299H)	c.549_550delGT (p.V184fsX8) *	OCA1
D	F	4	White	White	Gray	Positive	*TYR*	c.985T>C(S329P)	c.832C>T(R278X)	OCA1

* A novel mutation.

### Mutations identification and analysis

**Fig 2:**
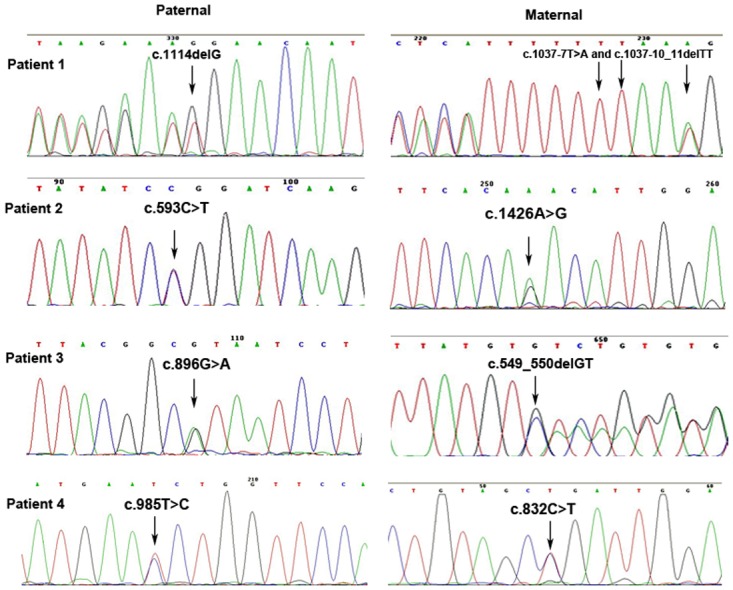
Sequencing results of *TYR* gene (patient A,C and D)or *OCA2* gene(patient B). Changes also seen in the father are shown on the left, while those inherited from the mother are shown on the right.

**Fig 3:**
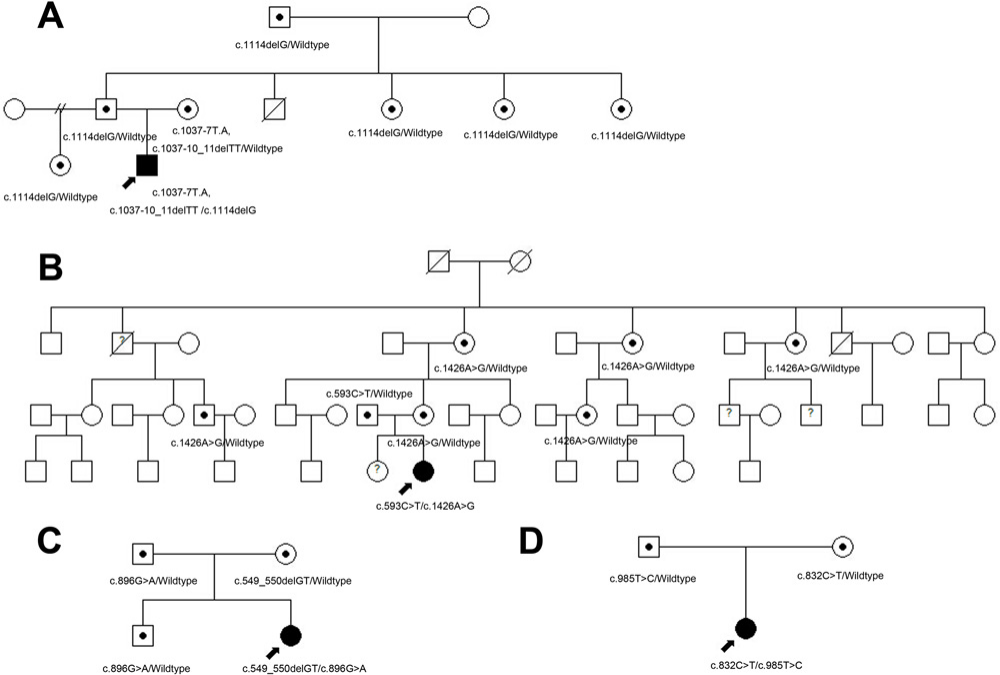
The pedigree of family A-D. A filled circle marked by an arrow indicates the probands and a dot in the middle of the circle indicates a carrier in the family. A double slash indicates divorce. A question mark in the middle of the circle or square indicates family members who were unavailable for clinical examination or DNA analysis. Square symbols denote males, the circles denote females.

**Fig 4:**
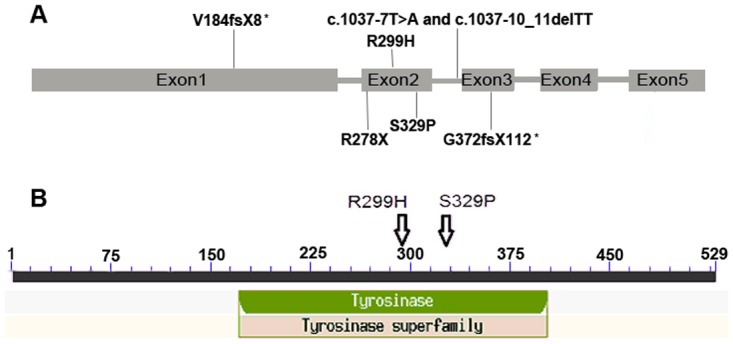
A summary of tyrosinase gene mutations identified in the present study. The novel mutations are marked by an asterisk. A: Exon structure of the *TYR* gene showing the positional mutations; B: Tyrosinase protein showing the common central domain of tyrosinase showing positions of missense mutations.

Sequencing of the relevant PCR fragments in exons of the *TYR* and *OCA2* genes revealed that all patients were compound heterozygotes (Fig 2). Patient A was heterozygous for c.1114delG, c.1037-7T>A and c.1037-10_11delTT changes in the *TYR* gene. The father and some family members (Fig 3A) who carried the c.1114delG (p.G372fsX112) allele also presented with blond or brown hair and white skin when they were born, but gradually accumulated pigment in both with age. However, the color of the iris and retina of these carriers appeared normal, even during childhood. Patient B was compound heterozygous for c.593C>T (p.P198L) and c.1426A>G (p.N476D) changes in the *OCA2* gene. Also, family members heterozygous for the p.N476D change (Fig 3B) showed with light hair and skin. Patient C was compound heterozygous for c.549_550delGT (p.V184fsX8) and c.896G>A (p.R299H) changes in the *TYR* gene while patient D was also compound heterozygous for c.832C>T (R278X) and c.985T>C (S329P) changes in the *TYR* gene (Table 3). The two novel mutations of the *TYR* gene (c.549_550delGT and c.1114delG) were not detected in any of the 105 normal controls (210 alleles) enrolled in this study. A summary of tyrosinase gene mutations identified in the present study and their positions in the *TYR* gene is shown in the Fig 4A. Positions of missense mutations in the common central domain of tyrosinase are shown in the Fig 4B. Position of the tyrosinase central domain is from http://www.ncbi.nlm.nih.gov/Structure/cdd/wrpsb.cgi?INPUT_TYPE=live&SEQUENCE=AGV39210.1.

## Discussion

The common disease-causing genes of OCA are the tyrosinase encoding gene *TYR* and *OCA2* which encodes the human homologue of the mouse p (pink-eyed dilution) gene. Tyrosinase is located on chromosome 11q14-21 and consists of 5 exons coding for a copper binding protein with 529 amino acid residue. Tyrosinase plays an important role in melanin biosynthesis, catalyzing the rate-limiting steps that convert L-tyrosine to L-DOPA and then to DOPAquinone[14]. *OCA2* is located on chromosome 15q11.2-q12 and its protein product, known as the *P* protein (NP_000266.2), has 838 amino acid residues. It is a transmembrane protein found in the melanosomal membrane [15–17]. Some studies reported that *P* protein may play a role in regulating the pH of melanosomes[18]. So far more than 200 mutations in the *TYR* gene and around 100 mutations in the *OCA2* gene are identified in different populations with OCA (http://www.hgmd.org/), but relatively fewer in Chinese patients[19].

In our study, we identified compound heterozygous mutations in the *TYR* or *OCA2* in four Chinese individuals by direct sequencing. The heterozygous mutations c.1037-7T>A and c.1037-10_11delTT in the *TYR* gene identified in patient A were firstly reported in Korean and Japanese patients[20–22]. Maki Goto et al[20] demonstrated that the splice site mutation could induce abnormal mRNA splicing, which inserted 4 bases (ACAG) upstream from the common acceptor site of exon 3 and resulted in premature termination codon downstream. The other mutation c.1114delG was a novel one. It is not present in dbSNP (http://www.ncbi.nlm.nih.gov/dbvar), the 1000 Genomes Database (http://browser.1000genomes.org/index.html), or the HGMD Professional Database (https://portal.biobase-international.com/hgmd/pro/search_gene.php). A single base deletion of G at position 1114 caused a frameshift alteration after codon 372 and then a premature termination at codon 484, resulting in a truncated protein. Patient B was compound heterozygous for c.593C>T (p.P198L) and c.1426A>G (p.N476D) changes in the *OCA2* gene. Suzuki T et al[23] identified the P198L allele in Japan for the first time. In 2007, Li et al[24] reported that prenatal diagnosis was performed in two Chinese families with OCA2 type. In their studies, p.N476D was one of the novel mutations in the *OCA2* gene and carriers were not noted to have any clinical signs or symptoms. However, in our studies, the individuals heterozygous for the p.N476D mutation presented with light hair and skin that darkened with age (data not shown), while the ocular features of albinism were absent. Carriers of c.1114delG showed similar findings. Patient C was compound heterozygous for c.549_550delGT and c.896G>A mutations in the *TYR* gene. To the best of our knowledge, c.549_550delGT in the *TYR* gene was also a novel mutation. It was not present in the HGMD or HGMD Professional Database, dbSNP, or the 1000 Genomes database. The variant c.549_550delGT caused a frameshift alteration after codon 183 (Valine) leading to a premature termination codon (PTC) which located at codon 192. And the c.896G>A (p.R299H) mutation was the most frequent allele in Chinese patients[19, 25], also found in Caucasians[26], Arab Christians[27, 28] and Koreans[29]. Patient D was compound heterozygous in the *TYR* gene for c.832C>T (p.R278X) and c.985T>C (p.S329P). The variant c.832C>T caused a premature stop codon at 278, found in several ethnic groups[25, 30–33]; the c.985T>C mutation was first reported in German individual in 2004[34].

Most types of OCA are inherited as an autosomal recessive trait. The patient’s parents are normally asymptomatic as pathologic mutation in one copy of the *TYR* or *OCA2* gene does not result in OCA. However, some individuals have been reported to have mild phenotype such as some degree of iris transillumination or hair and skin hypopigmentation in a heterozygous state [35]. In our study, carriers of two mutations (the c.1114delG in the *TYR* gene in family A and the c.1426A>G in the *OCA2* gene in family B) presented with a mild hypopigmentation phenotype, which was particularly obvious at a young age. The probands in the two families came to medical attention for nystagmus, not for their light coloration of hair and skin as the latter manifestation was thought by the family members to be a familial feature from each parent’s side. They didn’t realize the hypopigmentation represented OCA before molecular diagnoses. It thus seems likely that these two mutations (the c.1114delG mutation in the *TYR* gene and the c.1426A>G mutation in the *OCA2* gene) may be responsible for partial clinical manifestations of OCA in heterozygous carriers. The mechanism for this is unclear. It seems somewhat unlikely that this is a true dominant-negative effect, but might represent a gain of a deleterious function by the mutant gene products, or simple haploinsufficiency due to other genetic background in these families.

Wei et al[36] investigated the frequency of digenic mutations in Chinese OCA patients. They found that 134 (72.8% in total) patients had 2 pathologic mutations on one locus and 5 (2.7% in total) patients had digenic mutations of different genes by further examination. It indicated that the *TYR*, *OCA2*, *TYRP1* and *SLC45A2* genes may play synergistic roles during melanin biosynthesis. In our study, compound heterozygous mutations of the *TYR* or *OCA2* genes were identified in four Chinese individuals. The mutations c.549_550delGT and c.1114delG in the *TYR* gene are two unreported alleles, which were not present in the control group. Two mutations (the c.1114delG allele in the *TYR* gene and the c.1426A>G allele in the *OCA2* gene) may play a role in formation of certain clinical manifestations of OCA in heterozygous carriers.

Recessive compound heterozygous form indicated the mutant alleles of both copy are at the different locations and compound heterozygosity reflects the diversity of the mutation type of OCA. Patients in the compound heterozygous form may present with less severe phenotype compared with the one in the homozygous state. It can be found in our study that four patients all presented with milder form of OCA.

In summary, we report four OCA families and the molecular basis of their disease were identified by PCR-sequencing of all coding exons of the *TYR* and *OCA2* genes. This study expands the mutation spectrum of oculocutaneous albinism. It is the first report of the c.549_550delGT and c.1114delG mutations in the *TYR* gene in OCA1. In addition, two mutations (the c.1114delG in the *TYR* gene and the c.1426A>G in the *OCA2* gene) may be responsible for partial clinical manifestations of OCA in heterozygous carriers. Molecular genetic testing of *TYR*, *OCA2* and other relative genes is a useful tool for clinical diagnosis and genetic counseling of OCA.
